# Distribution of *Brugia malayi *larvae and DNA in vector and non-vector mosquitoes: implications for molecular diagnostics

**DOI:** 10.1186/1756-3305-2-56

**Published:** 2009-11-17

**Authors:** Sara M Erickson, Kerstin Fischer, Gary J Weil, Bruce M Christensen, Peter U Fischer

**Affiliations:** 1 Department of Pathobiological Sciences, University of Wisconsin-Madison, Madison, Wisconsin, USA; 2 Department of Internal Medicine, Infectious Diseases Division, Washington University School of Medicine, St Louis, Missouri, USA

## Abstract

**Background:**

The purpose of this study was to extend prior studies of molecular detection of *Brugia malayi *DNA in vector (*Aedes aegypti- *Liverpool) and non-vector (*Culex pipiens*) mosquitoes at different times after ingestion of infected blood.

**Results:**

Parasite DNA was detected over a two week time course in 96% of pooled thoraces of vector mosquitoes. In contrast, parasite DNA was detected in only 24% of thorax pools from non-vectors; parasite DNA was detected in 56% of midgut pools and 47% of abdomen pools from non-vectors. Parasite DNA was detected in vectors in the head immediately after the blood meal and after 14 days. Parasite DNA was also detected in feces and excreta of the vector and non-vector mosquitoes which could potentially confound results obtained with field samples. However, co-housing experiments failed to demonstrate transfer of parasite DNA from infected to non-infected mosquitoes. Parasites were also visualized in mosquito tissues by immunohistololgy using an antibody to the recombinant filarial antigen Bm14. Parasite larvae were detected consistently after mf ingestion in *Ae. aegypti- *Liverpool. Infectious L3s were seen in the head, thorax and abdomen of vector mosquitoes 14 days after Mf ingestion. In contrast, parasites were only detected by histology shortly after the blood meal in *Cx. pipiens*, and these were not labeled by the antibody.

**Conclusion:**

This study provides new information on the distribution of filarial parasites and parasite DNA in vector and non-vector mosquitoes. This information should be useful for those involved in designing and interpreting molecular xenomonitoring studies.

## Background

Human lymphatic filiarasis (LF) is caused by the mosquito-borne filarial nematodes *Wuchereria bancrofti*, *Brugia malayi*, and *B. timori*. These parasites are currently targeted for elimination by the Global Program for the Elimination of Lymphatic Filariasis (GPELF), and workers in this program have reported both achievements and future challenges to eliminating parasite transmission in endemic areas [1-3]. One important component of the elimination program is the ability to estimate infection prevalence and transmission rates, especially during mass drug administration (MDA), in order to accurately evaluate the progress towards the goal of LF transmission interruption [4]. Molecular detection assays provide sensitive and specific tools for identifying and distinguishing parasites in host populations. Molecular techniques commonly used to study LF infection, or exposure, in humans include the detection of parasite DNA, circulating filarial antigen, and filarial antibodies in blood samples [5]. Molecular techniques also have been applied to the detection of filarial worms in mosquitoes, and these primarily target parasite DNA [6-9].

The detection of parasite DNA in mosquito samples is a valuable tool for molecular xenomonitoring (MX), but this does not differentiate parasite developmental stages or distinguish whether the DNA is from living or dead parasites [10-12]. Recently, RNA-based assays have been developed to detect *B. malayi *and *W. bancrofti *in mosquitoes [13,14], including the distinction of *B. malayi *infected (a constitutive parasite transcript) and infective mosquitoes (a L3-specific transcript) [14]. However, RNA-based detection assays have not yet been tested in the field or incorporated into LF surveillance programs. Vector-parasite interactions influence the applicability and interpretation of molecular detection assays used in vector surveillance studies. There are several factors that should be carefully considered when using molecular techniques to investigate parasites within the mosquito intermediate host, including the (1) various life cycle stages and their tissue locations, (2) likelihood of parasite development to the infective stage, i.e., vector competence, and (3) limitations of the particular detection assay, i.e., ability to distinguish infection stages and living from dead parasites. The separation of mosquitoes into body regions has been used to circumvent the inability of some assays to distinguish infective-stage parasites. For example, *Anopheles *spp. have been divided into two body regions (head/thorax and abdomen) to provide better estimates of mosquitoes infected with *Plasmodium *sporozoites and/or pre-sporozoite stages [15-17] and the heads of blackflies have been removed (by mass dissection techniques) for the restricted, head-only, PCR assays targeting *Onchocerca *DNA, which is more likely to provide a better estimate of infective-stage parasites because other developmental stages generally reside outside of the head [18,19].

The studies conducted herein follow our previous work, which demonstrated that DNA-based diagnostics are unable to distinguish the developmental stage of LF parasites or whether parasites are living or dead in the mosquito [10]. Despite these limitations, there are benefits to using DNA-based assays over dissection to assess the persistence of filariasis in populations. Because filarial DNA is detectible for two weeks or longer following a microfilaremic blood meal in both vector and non-vector mosquitoes, all anthropophilic mosquitoes can be included in the screening of mosquitoes for parasite DNA to provide MX data [10]. Herein, we have further examined the persistence of filarial parasites and parasite DNA in mosquitoes; we used a combination of mosquito dissection, immunohistology and PCR assays to determine the location(s) of filarial worms and DNA in mosquitoes that are susceptible or refractory to filarial parasite development. These studies allowed us to assess the potential value of tissue specific assays (e.g., mosquito heads only) to estimate the prevalence of infective-stage larvae in mosquitoes; we also investigated the issue of direct mosquito to mosquito transfer of parasite DNA that could confound MX studies.

## Results

### Development of *B. malayi *in *Ae. aegypti *and *Cx. pipiens*

Table 1 summarizes the recovery of parasites from dissected mosquitoes. In *Ae. aegypti*-LVP, 73.6% of the recovered *B. malayi *mf at 2 h post ingestion (PI) had successfully penetrated the midgut, with 49.4% located in the thorax. At 14 days post ingestion of microfilaremic blood (DPI), 80% of *Ae. aegypti *harbored L3s. In contrast, from *Cx. pipiens *only mf were recovered and 93% of them were found in the midgut lumen.

**Table 1:** Distribution of *B. malayi *larvae in vector and non-vector mosquitoes as assessed by dissection.

	Time post ingestion	Number of parasites recovered^a^				Total worms^a^	Percentage of mosquitoes harboring parasites
				
		Midgut	Abdomen	Thorax	Head		
*Ae. aegypti*-LVP	2 h	23	21	43	n.d.	87	93% (6.2 ± 6.1)^b^
	7 d	0	0	65	0	65	73% (5.9 ± 5.9)
	14 d	n.d.	7	30	22	59 (20)	80% (3.9 ± 3.2)

*Cx. pipiens*	2 h	100	4	0	n.d.	104	93% (7.5 ± 7.1)
	7 d	0	4	0	0	4	13% (2.0 ± 1.4)
	14 d	0 (10)	0	0	0	0	0%

^a^Parasites were observed in mosquito dissections by microscopy at various times after ingestion of microfilaremic blood. Fifteen mosquitoes were dissected at each time point, except as noted (n.d., 10, or 20).

^b^Mosquito infection rate (mean intensity ± SD)

### *B. malayi *DNA detection in pooled mosquito body regions

**Figure 1:**
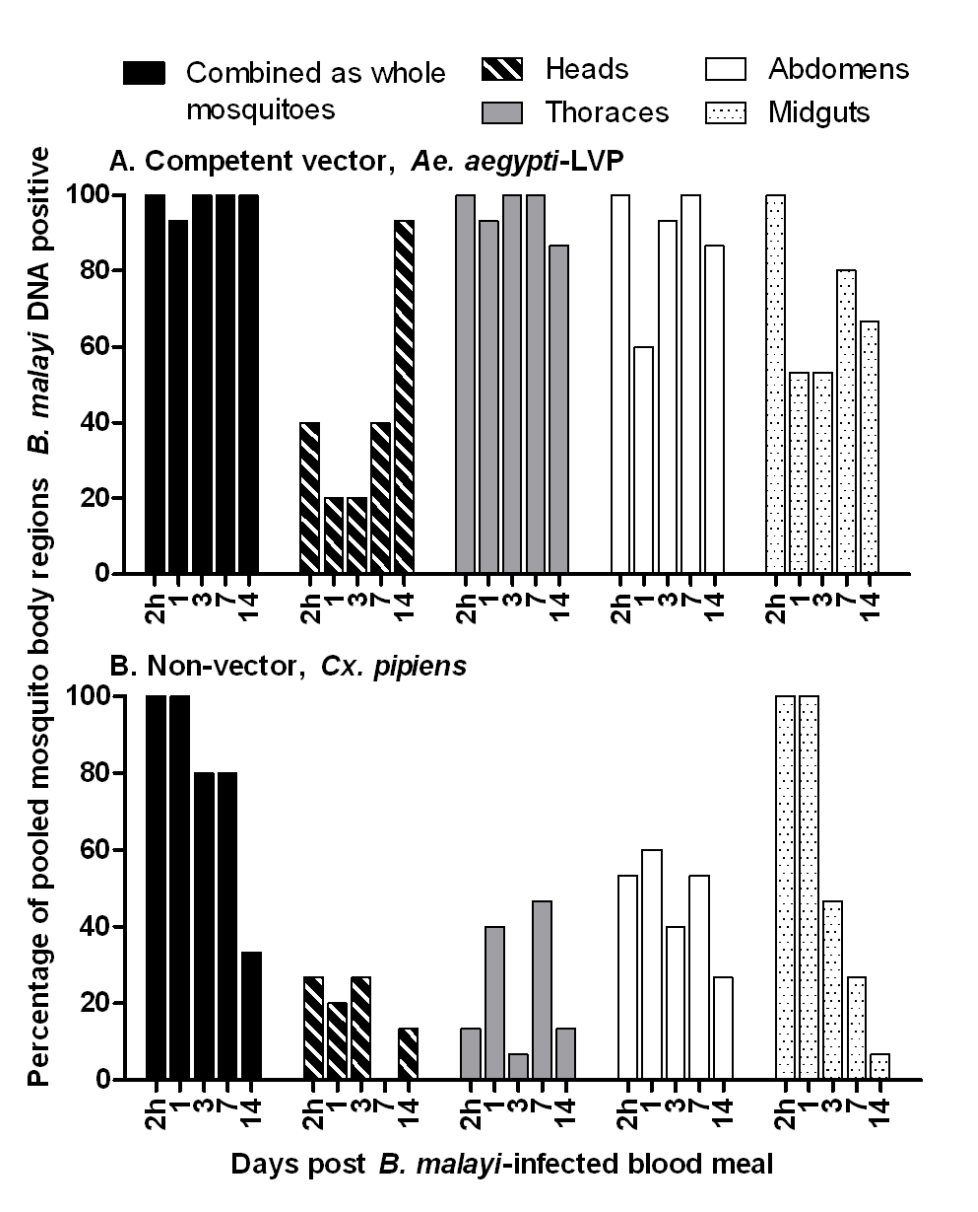
**Prevalence of *B. malayi *DNA in pooled samples of experimentally infected mosquitoes by body part (head, thorax, abdomen, and midgut) at different times post ingestion of microfilaremic blood**. **A ***Ae. aegypti*-LVP, competent *B. malayi *vector **B ***Cx. pipiens*, *B. malayi *non-vector.

PCR results are summarized in Figure 1. Parasite DNA was detected in 74% of *Ae. aegypti*-LVP pooled body region samples (n = 300) and 36% of *Cx. pipiens *(n = 300) pooled body regions tested. These differences were highly significant (*P *< 0.0001). In *Ae. aegypti*-LVP, parasite DNA was detected in all four body regions with 43, 71, 88, and 96% of heads, midguts, abdomens, and thoraces (n = 75 for each) positive by qPCR, respectively. Parasite DNA also was detected in all *Cx. pipiens *body regions, with 17, 24, 47, and 56% of heads, thoraces, abdomens, and midguts (n = 75 for each) positive, respectively. The differences in the percentage of *B. malayi *DNA positive samples were significant between mosquito species in all body regions (heads, *P *= 0.001; thoraces, *P *< 0.0001; abdomens, *P *< 0.0001) except the midguts (*P *= 0.09). The detection of parasite DNA within certain mosquito body regions was positively or negatively correlated with time. Specifically, the detection of *B. malayi *DNA was negatively correlated with time in 'whole body' *Cx. pipiens *(r^2 ^= 0.93, *P *= 0.0075) and *Cx. pipiens *midguts (r^2 ^= 0.82, *P *= 0.034), and positively correlated with time in *Ae. aegypti*-LVP heads (r^2 ^= 0.77, *P *= 0.05).

### *B. malayi *DNA detection in individual mosquito body regions

**Figure 2:**
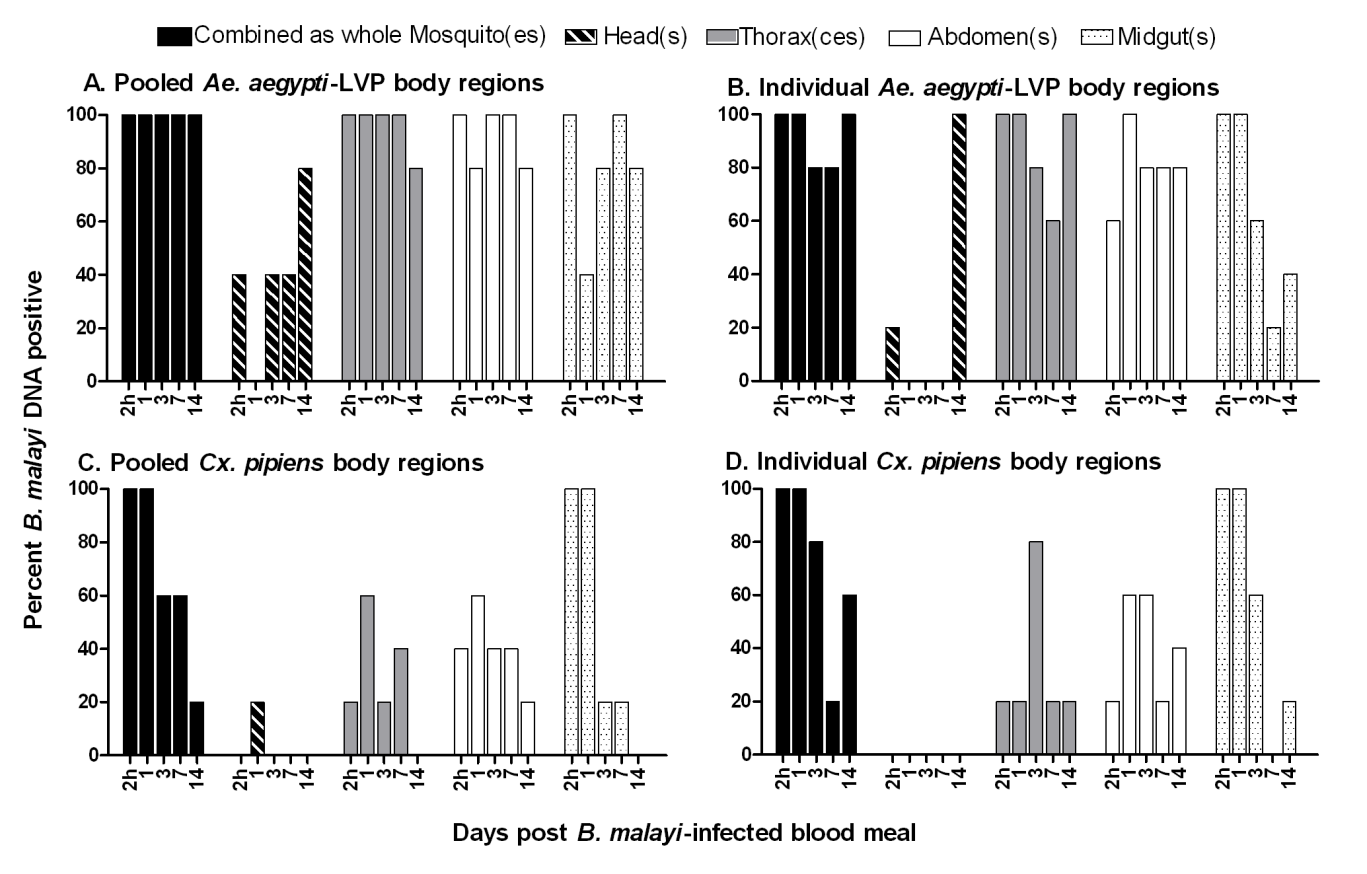
**Comparison of *B. malayi *DNA prevalence in pooled and individually tested mosquito body parts**. **A ***Ae. aegypti*-LVP body parts were tested in pools of four. **B ***Ae. aegypti*-LVP body parts individually tested for parasite DNA. **C ***Cx. pipiens *body parts tested in pools of four. **D ***Cx. pipiens *body parts individually tested for *B. malayi *DNA.

Individual mosquitoes that were separated into body regions for DNA detection assays were compared to results of the pooled mosquito body regions (Fig. 2). Following a microfilaremic blood meal of 191 mf/20 μl blood, there was no difference in DNA detection between pooled and individual *Ae*. *aegypti*-LVP body regions (*P*-values = 0.35-0.61) or between pooled and individual *Cx. pipiens *body regions (*P*-values = 0.78-1.0).

### Detection of parasite DNA in mosquito excreta and feces

**Figure 3:**
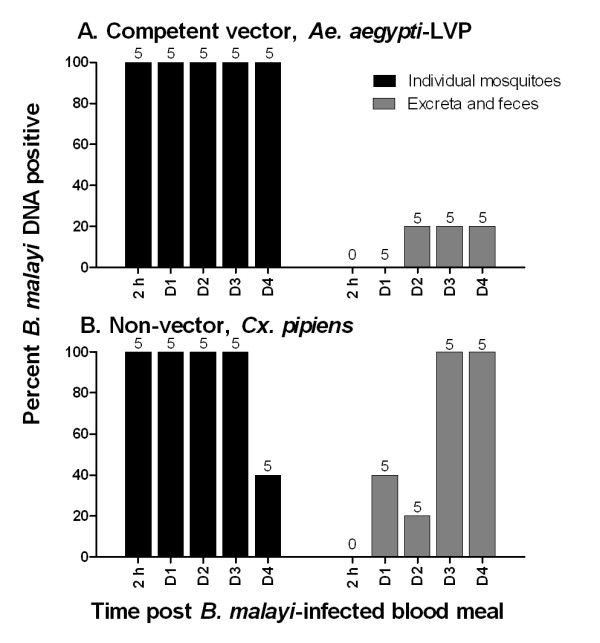
**Detection of *B. malayi *DNA in mosquito feces**. **A **Prevalence of parasite DNA in individually housed *Ae. aegypti*-LVP and their feces. **B **Prevalence of parasite DNA in individually housed *Cx. pipiens *and their feces. Sample number is indicated above each bar.

Figure 3 summarizes the detection of *B. malayi *DNA in individual housed mosquitoes and their voided excreta and feces. All mosquitoes were positive for parasite DNA immediately (2 hr) after ingesting microfilaremic blood. From 1-4 DPI all *Ae*. *aegypti*-LVP tested positive for parasite DNA, and three of these (15%) mosquitoes had detectible *B. malayi *DNA in their feces. In contrast, 60% of *Cx. pipiens *were DNA negative at 4 DPI, but *B. malayi *DNA was detected in 100% of *Cx. pipiens *feces tested at 3-4 DPI. Of the twenty samples of feces collected over the entire observation period of 1-4 DPI from each species, *B. malayi *DNA was detected in 15 and 65% of *Ae. aegypti*-LVP and *Cx. pipiens *fecal samples, respectively (*P *= 0.003).

### Parasite DNA contamination of *B. malayi *positive and negative mosquitoes

In order to test the possibility that infected mosquitoes contaminate uninfected mosquitoes while they are together in the same trap or sampling tube, we housed uninfected *Ae. aegypti*-LVP together with *Cx. pipiens *that had fed on a microfilaremic gerbil. After 7 days, mosquitoes were collected and pooled by species. None of the 17 *Ae. aegypti*-LVP pools (with 10 mosquitoes per pool) were positive by real-time PCR. In contrast, 17 of the 21 *Cx. pipiens *pools (with 5 mosquitoes each) were positive. Most of these samples had relatively high Ct values indicating small amounts of *B. malayi *DNA, but 5 pools had higher Ct values ranging between 29 and 35. Although *B. malayi *DNA can be detected in feces of infected mosquitoes, feces did not cause false positive DNA signals from uninfected mosquitoes after co-housing.

### Detection of Bm14 in mosquito-stage parasites by immunohistology

**Figure 4:**
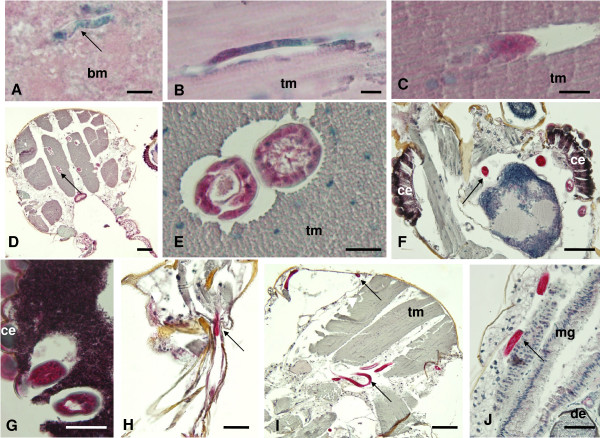
**Immunohistological detection of *B. malayi *larvae in *Ae. aegypti*-LVP using polyclonal antibody to recombinant antigen Bm14**. **A **Non-stained mf (arrow) in the midgut at 1 DPI: **B **Longitudinal section of a stained L1 in the thorax muscles at 1 DPI: **C **Cross-section of a stained larva at 3 DPI: **D **Multiple cross-sections of strongly labeled L2s (arrow) at 7 DPI in the thorax. **E **Cross-sections of two strongly labeled L2s with body cavity and developing intestine at 7 DPI: **F **Overview of the head with 4 cross-sections of strongly labeled L3s (arrow) at 14 DPI: **G **Cross-section of 2 well stained larvae close to the mosquito eye. **H **Labeled L3 at 14 DPI in the mosquito mouthparts. **I **Multiple strongly labeled L3s (arrows) in the thorax. **J **Strongly labeled L3 in the abdomen. (bm, blood meal; ce, compound eye; de, developing egg; tm, thorax muscles; mg, midgut; um, uterus membrane). Scale bars: A-C, E 25 μm; D, F-J 50 μm.

Immunohistology studies were performed to confirm the dissection results and to better document the fate of *B. malayi *in vector and non-vector mosquitoes. In *Ae. aegypti-*LVP, unlabeled mf were detected within the midgut directly after the bloodmeal (Fig. 4A). Strong-labeling was observed with the Bm14 antibody after the larvae reached the thoracic muscles. Thus this antibody can be used to sensitively detect developing filarial larvae in vectors (Fig. 4B-E). Strongly labeled L3s were observed in all body parts of *Ae. aegypti*-LVP at 14 DPI (Fig. 4F-G). L3s were not confined to the head or the thoracic musculature; they were also seen in the abdomen, outside of the midgut (Fig. 4J).

**Figure 5:**
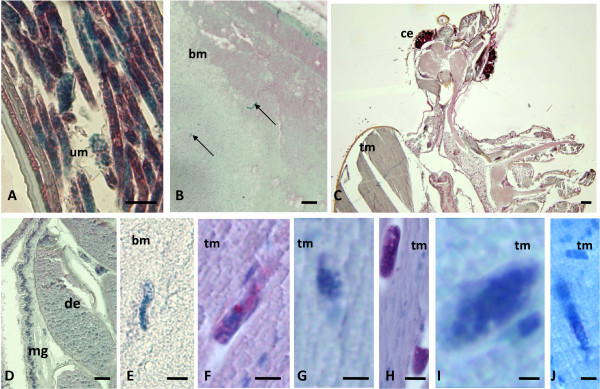
**Immunohistological detection of *B. malayi *larvae in the uterus of adult *B. malayi *or in experimentally infected mosquitoes using polyclonal antibody to recombinant antigen Bm14**. **A **The antibody labeled intra-uterine, stretched mf in an adult female *B. malayi *worm. **B **Multiple unlabeled mf in the midgut of *Cx. pipiens *at 2 h PI: **C **Overview of the head and parts of the thorax at 14 DPI negative for *B. malayi *larvae. **D **Abdomen of *Cx. pipiens *at 3 DPI without any visible developing *B. malayi *larvae (labeled or unlabeled). **E **Magnified, unlabeled mf in the midgut of *Cx. pipiens *at 2 h PI: **F **Section of a labeled L1 in the thorax muscles of *Ae. aegypti*-LVP at 1 DPI: **G **Section of an unlabeled L1 in the thorax of *Ae. aegypti*-RKF at 1 DPI: **H **Two sections of labeled larvae in the thorax of *Ae. aegypti*-LVP at 3 DPI: **I **Section of an unlabeled larva in *Ae. aegypti*-RKF at 3 DPI. **J **Section of an unlabeled larva in *Ae. aegypti*-RKF at 7 DPI (compare Fig. 4D). Scale bars: A-D 25 μm; E-F 10 μm.

Stretched, intrauterine mf in adult female *B. malayi *are usually labeled by the Bm14 antibody (Fig. 5A), but mf were not labeled in the midgut of *Cx. pipiens *directly after the blood meal. No larvae were detected in histological sections of *Cx. pipiens *at later times points (Fig. 5C, D). Dead and/or dying larvae in the thorax of *Brugia *refractory *Ae. aegypti*-RKF were not labeled by Bm14 (Fig. 5G, I, J). In contrast, developing larvae in *Ae. aegypti*-LVP were always strongly labeled at the same time points (Fig. 5F, H). These results suggest that the anti-Bm14 antibody specifically detects viable and developing *B. malayi *larvae in vectors.

## Discussion

The application of molecular assays to detect parasites within vectors is influenced by the vector-parasite interaction. In these studies, we analyzed the detection of filarial worms in susceptible and refractory mosquitoes by targeting parasite DNA or protein in molecular assays. The three mosquito strains examined have very different interactions with *B. malayi *that were documented in our previous paper on detection of parasite DNA from living and dead parasites within mosquitoes [10]. The follow-up studies discussed herein were designed to investigate: (1) the mosquito body region(s) containing the persistently detected parasite DNA within *Cx. pipiens *(in this mosquito species mf are seldom able to migrate out of the midgut lumen), and (2) the detection of parasite proteins as larvae develop in susceptible and refractory mosquitoes.

Separation of mosquitoes into body regions (head, thorax, abdomen, and midgut) prior to PCR assays provided further details on the location of detectible parasite DNA within mosquitoes. In both vector *Ae. aegypti*-LVP and non-vector *Cx. pipiens*, the head region had the lowest parasite DNA detection rates (43 and 17% respectively) when all time points are considered. However, there was a striking difference at 14 days when nearly 100% of pools of *Ae. aegypti*-LVP heads and very few pools of *Cx. pipiens *heads contained parasite DNA. This finding correlates well with infectivity rates at that time point. Although DNA detection in mosquito heads might provide a better estimate of infectivity rates than DNA detection in whole mosquitoes (Fig. 2), we do not advocate separating heads for this purpose in field studies, because false positive and false negative infectivity signals are likely to be high with this approach. Because PCR can detect DNA from live or dead parasites from any developmental stage, heads could be falsely positive (without L3s being present) because of remnants of ingested mf (especially in mosquitoes with armed cibarial and/or pharyngeal pumps). Heads could provide a false negative infectivity signal when mosquitoes contain L3s in other body parts. Our results and prior studies [20] have shown that L3s are not restricted to the head.

*B. malayi *DNA also was not restricted to the midgut in *Cx. pipiens*. Although most mf do not leave the *Cx. pipiens *midgut lumen, mf are sometimes detected outside of the midgut by dissection (Table 1 and Erickson and Christensen, unpublished data). Although these few mf that do penetrate the midgut epithelium could be the source of parasite DNA outside of the *Cx. pipiens *midgut, an alternative explanation was also examined. In compatible vectors, the majority of mf penetrate the midgut epithelium very quickly (within 1.5 h) after ingestion [21], and those that are unable to penetrate remain within the midgut lumen and are destined for digestion and/or defecation. This is in agreement with our observation that at 2 h PI almost 50% of mf were recovered from the thorax. In parasite-vector pairs that limit or prevent mf penetration of the mosquito midgut (e.g., *Brugia*--*Cx. pipiens *and certain *Wuchereria*--*Culex *spp. interactions) [22-25], parasite material is likely to be shed in mosquito feces. To test the hypothesis that DNA detected outside of the midgut is due to parasite DNA on exterior mosquito surfaces, excreta and feces from *Ae. aegypti *and *Cx. pipiens *were examined for parasite DNA. *B. malayi *DNA was detected in voided materials of both species, but significantly more *Cx. pipiens *had detectible parasite DNA in their excreta and feces following a microfilaremic blood meal (Fig 3). Although external contamination could cause false positive PCR results, our mosquito co-housing results suggest that cross-contamination is probably uncommon.

However, to prevent cross-contamination, the following steps are recommended for processing wild-caught mosquitoes: (1) Immediately kill mosquitoes to limit mosquito-mosquito contact, especially if blood engorged mosquitoes are present; (2) When killing mosquitoes, avoid methods of slow death in which the mosquito host is killed and parasites are not, because it is possible for L3s to escape the body of living, dead, and dying mosquitoes, especially if mosquitoes come in contact with aqueous fluids [26-28]; (3) Pool mosquitoes as soon as possible after collection; (4) When sorting mosquitoes into pools, tools such as forceps and brushes should not pierce the mosquito body; and (5) Mosquito sampling should be restricted to whole females (head, thorax and abdomen intact). This will reduce error that could be introduced by testing random body parts from unidentified individuals.

Immunohistochemistry provides an alternative approach to detecting and studying parasite migration and development in mosquitoes; several studies have used this approach with antibodies to *Plasmodium *circumsporozoite protein (CSP) [16,29-31]. Most immunodiagnostic research on filariasis has focused on the parasite stages that occur within the vertebrate host [32,33], and this work has produced sensitive and specific diagnostic tests for Bancroftian and brugian filariasis [5,34,35]. In contrast, few studies have examined antigen detection as a method for identifying and distinguishing LF parasites in mosquitoes. A monoclonal antibody raised against *B. malayi *L3s (NEB-D_1_E_5_) is specific to a *B. malayi *L3 surface antigen and distinguishes *B. malayi *L3s from infective-stage larvae of other filarial worms apart from *B. timori *[36,37]. In the current study, antibodies to recombinant *B. malayi *antigen Bm14 [38,39] were used to detect this protein in filarial larvae in mosquitoes. Bm14 was detected in mf and all other developmental stages of parasites in the competent vector, *Ae. aegypti*-LVP. In contrast, the protein was not detected in parasites present in non-vector *Cx. pipiens *(harboring mf in the midgut), or *Ae. aegypti*-RKF (harboring parasites that developmentally arrest as L1s which then die within mosquito muscle cells). These results suggest that Bm14 may be a specific biomarker for viable filarial parasites in mosquitoes.

## Conclusion

Improved methods are needed for assessing changes in mosquito infection and infectivity rates in the context of LF control/elimination programs. As infection rates in humans and vectors decrease following MDA, increased numbers of mosquitoes must be tested to accurately estimate parasite prevalence [40]. This makes dissection impractical, and favors use of molecular detection assays with pooled mosquitoes. This study provides new information on the persistence of filarial worm DNA in non-vectors that has practical implications for MX studies regarding methods for processing field-caught mosquitoes and for interpreting MX data. Additional studies are needed to determine whether the presence of Bm14 antigen is a reliable marker for viable filarial worms in pooled mosquito samples. Although this study focused on *B. malayi*, the findings may be of interest to scientists and programs that use molecular techniques to detect other pathogens (helminths, viruses, or protozoa) in vectors.

## Materials and methods

### Mosquito maintenance and parasite exposures

Mosquitoes used for these studies were obtained from colonies of *Aedes aegypti *(black-eyed, Liverpool strain; LVP), *Ae. aegypti *(Rockefeller strain; RKF) and *Culex pipiens pipiens (*Iowa strain) maintained at the University of Wisconsin-Madison, as previously described [21,41]. These mosquitoes differ in their vector competence for *B. malayi*. *Ae. aegypti*-LVP support the development of *B. malayi *from mf to L3s, but parasites do not develop in *Cx. pipiens*, because mf do not penetrate the midgut epithelium. In *Ae. aegypti*-RKF, mf penetrate the mosquito midgut and migrate into thoracic muscles where they fail to develop to L2s. This mosquito strain was only used for comparison in the immunohistology experiments. Four- to seven-day-old mosquitoes were sucrose starved ~14 h prior to blood feeding. Mosquitoes were exposed to *B. malayi *by blood feeding on microfilaremic cat blood in a water-jacketed membrane feeder fitted with a parafilm membrane [42]. Mosquitoes also were blood fed on uninfected gerbils (*Meriones unguiculatus*) to serve as parasite-negative, blood-fed controls. Engorged mosquitoes were sorted and maintained in the laboratory.

Laboratory animals were handled according to guidelines approved by the Animal Care Committee at the University of Wisconsin-Madison. The mf densities of *B. malayi*-infected cat blood obtained from the NIAID Filariasis Research Reagent Repository Center http://www.filariasiscenter.org used in these studies ranged from 24-191 mf/20 μl blood.

### Mosquito dissection

Five mosquitoes were dissected at 2 hr, 7 d, and 14 d post ingestion of microfilaremic blood (PI) to estimate the mean intensity of infection, and to record the stage of *B. malayi *development. Individual mosquitoes were separated into head, thorax, midgut, and abdomen, and each body region was teased apart and individually examined for parasites by microscopy as previously described [10].

*Ae*. *aegypti*-LVP and *Cx. pipiens *were separated into body regions (head, thorax, midgut, and abdomen) and placed, separately or in pools of four, into 2.0 ml microcentrifuge tubes for parasite DNA detection. To create a pooled sample, four mosquitoes were separated into body regions, and the body regions were combined by type into tubes. For example, four mosquitoes were used to produce one pool of four heads, one pool of four thoraces, one pool of four abdomens, and one pool of four midguts. Five pooled samples were prepared at 2 h, 1, 3, 7, and 14 d PI; thus, a total of 20 mosquitoes were collected at each time point for pooled samples. In addition to creating pooled samples, individual mosquitoes were dissected into body regions as described above and then placed individually into tubes. Five individuals were dissected at each time point to create twenty samples: five tubes contained individual heads, five contained a single thorax, five contained an abdomen, and five contained a midgut. These samples were screened for *B. malayi *DNA to compare detection results between individuals and pooled samples. All samples were cataloged to track a given body region back to the particular mosquito or pool of mosquitoes, allowing the assay results from each body region to be combined into results for the 'whole mosquito.' For example, if at least one of the body region samples was positive; then the 'whole mosquito' was considered positive and only if all body regions tested negative; then the 'whole mosquito' was considered negative.

### Collection of excreta and feces from individual mosquitoes

To examine possible sources of parasite DNA contamination, mosquitoes were individually housed to collect material voided by excretion (excreta processed from Malphigian tubules) and defecation (feces containing undigested material from the midgut) [43]. Immediately following blood engorgement, mosquitoes were individually housed in 2.0 ml microcentrifuge tubes. Mosquitoes were maintained for up to 4 DPI by providing 10% sucrose solution in drops on the mesh-screen top fitted on each tube. At 24 h intervals, mosquitoes and their voided matter were collected for DNA extraction. First, the mosquito was removed from the collection tube, transferred to a clean microcentrifuge tube and flash frozen in dry ice. Then, the collection tube containing the excreta and/or feces was labeled and also frozen. All samples were stored at -80°C until DNA extraction. Five mosquitoes of each species were collected at 2 h and 1-4 d PI, and voided material was sampled at 1-4 DPI. In addition, the appearance of a blood bolus was recorded for each mosquito to provide data on the presence or absence of blood meal remnants within the mosquito midgut. Mosquitoes that blood fed on uninfected gerbils also were housed individually and sampled at each time point for negative controls.

### Co-housing studies to determine whether *B. malayi *DNA is transferred from infected to uninfected mosquitoes

Uninfected *Ae aegypti *were housed together with *B. malayi*-infected *Cx. pipiens *that had fed on two *B. malayi*-infected gerbils with microfilaremias of 68 and 146 mf/20 μl blood. Engorged *Cx. pipiens *were sorted from non-blood fed mosquitoes, and the engorged mosquitoes were mixed with non-blood fed *Ae. aegypti*. Four cartons were studied with each containing ~60 uninfected *Ae. aegypti *and ~40 *B. malayi*-infected *Cx. pipiens*. At 7 DPI, mosquitoes were collected for qPCR detection of parasite DNA. Cold anesthetized mosquitoes were sorted by species and placed in pools of five individuals for *Cx. pipiens *and pools of ten individuals for *Ae. aegypti*. Mosquitoes were flash frozen on dry ice and stored at -80°C.

### DNA extraction and detection

Genomic DNA from pooled mosquitoes or body parts was extracted using a commercial column method as described previously [10]. Quantitative real-time PCR was performed using an MGB probe to detect a 120 bp fragment of the *Brugia Hha*I repeat [44]. In all real-time PCR assays, water was used as no-template negative control; DNA extracted from a pool of non-infected mosquitoes acted as extraction negative control, and 100 pg of DNA isolated from adult *B. malayi *was used as positive control.

### Immunohistology

Five mosquitoes of each species were collected at 2 h, 1, 3, 7, and 14 d PI (i.e., the same time points as DNA detection assays) and stored in 80% ethanol at room temperature until embedding. Mosquitoes were embedded in paraffin, and *B. malayi *larvae were stained using the alkaline phosphatase anti-alkaline phosphatase method as described previously [45]. A polyclonal mouse antibody raised against recombinant Bm14 protein was used as the primary antibody for these studies [38].

### Statistical Analysis

Data were graphed and analyzed with GraphPad Prism 5.0 http://www.graphpad.com. Fisher's exact tests, with two-tailed *P*-values, were used to compare parasite development and DNA detection between *Ae*. *aegypti*-LVP and *Cx*. *pipiens*. Pearson correlation tests were used to test for trends in parasite DNA detection over time. Statistical results were considered significant at *P *≤ 0.05.

## List of abbreviations

MF: microfilariae; L1, L2, L3: first- to third-stage larva; PI: post ingestion of microfilaremic blood; DPI: days post ingestion of microfilaremic blood; MX: molecular xenomonitoring.

## Competing interests

The authors declare that they have no competing interests.

## Authors' contributions

SE participated in the conception of the study including its design and organization, performed mosquito exposures and sample collection, data interpretation, statistical analysis, and drafted the manuscript. KF carried out the DNA detection and immunohistology assays, and data interpretation. GW participated in data interpretation and critical manuscript revisions. BC participated in study design, data interpretation, and critical manuscript revisions. PF participated in the conception of the study including its design and organization, data interpretation, and helped draft the manuscript. All authors read and approved the final manuscript.
